# Complete mitochondrial genome of *Histia rhodope* Cramer (Lepidoptera: Zygaenidae)

**DOI:** 10.1080/23802359.2017.1375876

**Published:** 2017-09-09

**Authors:** Shuying Peng, Wanrong Jia, Zhijun Huang, Yuchen Wang, Yan Li, Zhuoran Huang, Yunfang Zhang, Xu Zhang, Jianhua Ding, Xuexia Geng, Jun Li

**Affiliations:** College of Life Science, Huaibei Normal University, Huaibei, PR China

**Keywords:** Mitochondrial genome, *Histia rhodope* Cramer, lepidoptera, zygaenidae, mitogenome

## Abstract

*Histia rhodope* Cramer, found in India, Chinese, Burma, Indonesia and other Southeast Asian regions, belongs to Lepidoptera family Zygaenidae. In this study, we describe the genomic features of the mitogenome sequences of these insects. The mitogenome of *Histia rhodope* Cramer is 15,205 bp long consisting a typical set of genes (13 protein-coding genes, 22 tRNA genes and two rRNA genes) and one major 376 bp non-coding A + T-rich region. All PCGs of *Histia rhodope* Cramer start with ATN codons except *cox1* which start with CGA codon and all PCGs stop at TAA codons. Phylogenetic analysis demonstrates that *Histia rhodope* Cramer and *Rhodopsona rbiginosa* are clustered together into a monophyletic group Zygaenidae. Zygaenidae is phylogenetically closer to Limacodiae than Tortricidea.

*Histia rhodope* Cramer is belongs to Lepidoptera family Zygaenidae. They are harmful to plants of Bischofia and have been found in India, Chinese, Burma, Indonesia and other Southeast Asian regions. Mitogenomes have been involved in diverse studies of molecular evolution for its protein-coding genes (PCGs) sequence conservation, maternal inheritance and rapid evolution. And only one complete mitochondrial genome of Zygaenidae has been reported (Tang et al. 2014).

The specimens were obtained from Xiangshan mountain, Huaibei city, Anhui province, PR China (116°48′34″E, 33°59′1″N). We extracted the total DNA from the specimens as PCR template, designed PCR primers according to the conserve sequences of mitochondrial DNA of Lepidoptera insects, amplified the overlapping fragments using high fidelity Taq enzyme and ensure every fragments sequenced at least three times. These fragments were assembled into a complete linear mitochondria DNA sequence using the DNAStar package (DNAStar Inc. Madison, WI) and the mitogenome annotated using NCBI BLAST (http://blast.ncbi.nlm.nih.gov/Blast).

The complete mitogenome of *Histia rhodope* Cramer (GenBank accession number MF542357) is 15,205 bp and consists of 13 PCGs, 22 tRNAs, two rRNAs for the small and large subunits (rrnS and rrnL) and one AT-rich region (control region). All PCGs start with ATN codons except *cox1* which start with CGA codon and all PCGs stop at TAA codons.

To construct phylogenetic tree, we collected the complete mitochondrial DNA sequences of Limacodiae, Zygaenidae or Tortricidea from GenBank for these species are phylogenetically close to each other. Multiple sequence alignments were conducted using ClustalX 2.1 (Gene Codes Corporation, Ann Arbor, MI), and phylogenic trees were constructed by MEGA 7.0, using the Neighbour-Joining method with a bootstrap test of 1000 replications (Kumar et al. 2016). Two species of Lasiocampoidea were utilized as outgroup.

Phylogenetic analysis demonstrates that the monophyly of families Zygaenidae, Limacodiae or Tortricidea is well supported. *Histia rhodope* Cramer and *Rhodopsona rbiginosa* are clustered together into a monophyletic group Zygaenidae. Zygaenidae is phylogenetically closer to Limacodiae than Tortricidea (Figure 1).

**Figure 1.**
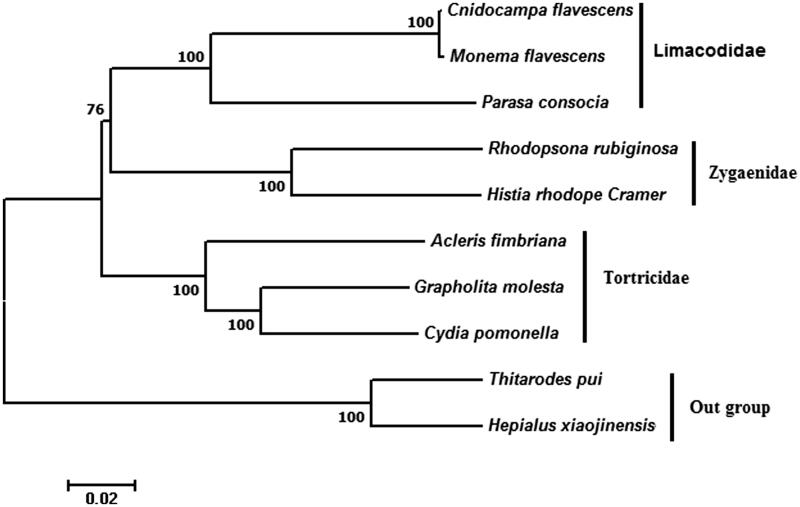
Phylogenetic tree constructed by the neighbour-joining (NJ) method with a bootstrap test of 1000 replications based on complete mitochondrial DNA sequences. Two species of Hepialoidea were utilized as outgroup. GenBank accession numbers are as follows: *Histia rhodope* Cramer MF542357 (the present study); *Cnidocampa flavescens* KY628213(unpublished); *Monema flavescens* KU946971 (Liu et al. 2016); *Parasa consocia* KX108765 (unpublished); *Rhodopsona rubiginosa* KM244668 (Tang et al. 2014); *Acleris fimbriana* HQ662522 (Zhao et al. 2016); *Grapholita molesta* HQ392511 (Gong et al. 2012); *Cydia pomonella* JX407107 (Shi et al. 2013); *Thitarodes pui* KF908880 (Yi et al. 2016); *Hepialus xiaojinensis* KT834973 (Chen et al. 2017).

## Depository

The specimens and the template DNA is respectively deposited in Specimens Room and in Human and Animal Genetics Laboratory, College of Life Science, Huaibei Normal University, Huaibei city, Anhui province, PR China.
